# Expanded Polycarbonate (EPC)—A New Generation of High-Temperature Engineering Bead Foams

**DOI:** 10.3390/polym12102314

**Published:** 2020-10-10

**Authors:** Nick Weingart, Daniel Raps, Justus Kuhnigk, Andreas Klein, Volker Altstädt

**Affiliations:** 1 Department of Polymer Engineering, University of Bayreuth, 95444 Bayreuth, Germany; nick.weingart@uni-bayreuth.de (N.W.); justus.kuhnigk@uni-bayreuth.de (J.K.); 2 Covestro Deutschland AG, 51365 Leverkusen, Germany; daniel.raps@covestro.com (D.R.); andreas.klein@covestro.com (A.K.)

**Keywords:** foaming, polycarbonate, particle foam, bead foam, expanded polycarbonate, EPC, EPP, EPET, mechanics, morphology

## Abstract

Bead foams serve in a wide variety of applications, from insulation and packaging to midsoles in shoes. However, the currently used materials are limited to somewhat low temperature or exhibit significant changes in modulus in the temperature range of many applications due to their glass transition. By comparison, polycarbonate (PC) exhibits almost constant mechanics for temperatures up to 130 °C. Therefore, it appears as an advantageous base material for bead foams. The aim of the publication is to provide comprehensive data on the properties of expanded PC (EPC) in comparison to already commercially available expanded polypropylene, EPP, and expanded polyethylene-terephthalate, EPET. A special focus is set on the thermo-mechanical properties as these are the most lacking features in current materials. In this frame, dynamic mechanical analysis, and tensile, bending, compression and impact tests at room temperature (RT), 80 °C, and 110 °C are conducted for the three materials of the same density. Already at RT, EPC exhibits superior mechanics compared to its peers, which becomes more pronounced toward higher temperature. This comes from the low sensitivity of properties to temperature as EPC is used below its glass transition. In summary, EPC proves to be an outstanding foam material over a broad range of temperatures for structural applications.

## 1. Introduction

Over the last decades, bead foams have become part of our daily life. The variety of applicational fields seems endless. From commodity usage as packaging or insulating thermoboxes [1] to high-performance applications in running shoes [2,3], polymer foams play an increasingly important role. Nevertheless, the usage fields are limited by the thermal stability of the thermoplastic material. Commodity foams like expanded PS (EPS) or expanded PP (EPP) are usually used for low to mid-temperature applications [4,5], while the variety of polymer foams for higher usage temperatures becomes significantly scarce, especially for engineering materials [6]. Only a few bead foam options exist for elevated temperature applications, i.e., expanded PET (EPET) distributed by the company Armacell [7]; expanded PBT (EPBT), a development of the department of Polymer Engineering [8]; several polyamide bead foams (EPA) (by Asahi Kasei [9], a PA 6 bead foam Ultramid^®^ by BASF SE [10], and a polyamide 12 (PA12) bead foam developed by the department of Polymer Engineering [11]), a SunForce^®^ modified polyphenyl ether (mPPE) bead foam by Asahi Kasei [12]; and a polyether sulfone (PESU) bead foam developed by BASF SE [13]. The newest high-temperature bead foam, which will be introduced in this work, is expanded polycarbonate (EPC), and was developed in collaboration with Covestro Deutschland AG and the department of Polymer Engineering. This novel bead foam has an amorphous structure and increased mechanical properties with analogies to polystyrene.

The first publication about foamed polycarbonate was published by Kumar et al., who described the production of batch-foamed polycarbonate (110 kg/m^3^) with CO_2_ as a blowing agent (BA) in the early 1990s [14], followed by the detailed cell growing mechanism 10 years later [15]. Furthermore, more research groups studied the discontinuous generated polycarbonate foams like Ma et al. [16], who produced microcellular PC-foam and investigated the foaming parameters, as well as Weller et al. [17] with comparable results and trends.

Compared to the discontinuous process, only a few publications have reported a continuous foaming method for polycarbonate. Park et al. [18] researched the foam extrusion of microcellular polycarbonate with a **filament-die** and the required processing parameters. They also investigated the expansion behavior, nucleation of CO_2_-loaded melt, and die-geometry-dependent pressure drop rates. The lowest achieved density was reported to be ~85 kg/m^3^ with 7 wt.% CO_2_ and a die temperature of 160 °C [18]. In addition, the working group of Gendron [19] reported a continuous extrusion of polycarbonate foams. They investigated the correlation between blowing agent concentration (CO_2_ and pentane) and foam density. Unlike Park et al., Gendron reported that it was impossible to solve more than 2–3 wt.% CO_2_ in polycarbonate under the given conditions (different setup). They succeeded in achieving the lowest foam density with 1.5 wt.% CO_2_ and an analog with 5 wt.% pentane. Higher blowing agent contents led to increased foam densities [19].

In terms of mechanical properties, commodity bead foams like EPS and EPP are already well-characterized at room temperature. The mechanical properties at elevated temperatures, on the other hand, are barely available in the literature, making this the first publication comparing temperature-dependent mechanical performance of bead foams. For PC-foams in particular, only a few publications discuss basic mechanical behavior (batch-foams) [20,21], but there is no literature available about PC-bead foams and its temperature-dependent mechanical performance. Therefore, this study will establish the compressive, tensile, bending, and impact behavior of EPC, as well as thermal conductivity at different temperatures (up to 110 °C) in comparison to the established commercially available bead foams like EPP and EPET.

Furthermore, a thorough review about existing literature on the topic of foamed polycarbonate is summarized in Table 1.

According to the literature survey, the common foaming window for polycarbonate in a batch-foaming setup is between 110 and 160 °C, resulting in typical foam densities around 100–450 kg/m^3^. Only a few papers exist for a continuous setup for bead foam production with CO_2_ as the blowing agent foamed at temperatures around 150–240 °C, resulting in foam densities at a magnitude of 400 kg/m^3^. 

## 2. Materials and Methods 

### 2.1. Materials

The polycarbonate used for this study was a development-grade CS-PCS-CVKKK-5-12 86538830 (Covestro Deutschland AG, Leverkusen, Germany). The provided material has an M_W_ of 31,000 g/mol (PDI of 2.8) and an MVR of 6.0 cm^3^/10 min (300 °C/1.2 kg). Additionally, an experimental modifier in low concentrations (below 1 wt.%) was used to increase the melt strength and the melt pressure. 

For comparison of the mechanical properties, commercially available EPP (Neopolen HD 130) (BASF SA, Ludwigshafen, Germany), as well as EPET (first generation of ArmaShape) (Armacell GmbH, Münster, Germany), was molded at a steam-chest-molding service provider (Bavaria, Germany) at material-optimized molding conditions (best part quality) to parts of 200 ± 10 kg/m^3^ and used for this characterization. 

### 2.2. Experimental

#### Bead Foaming

**Figure 1:**
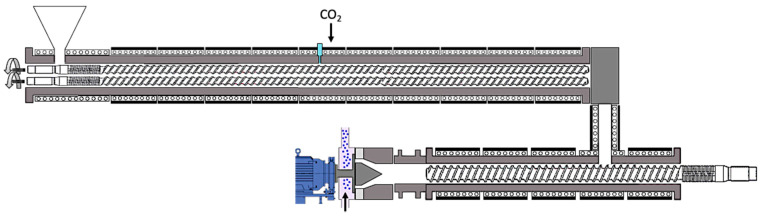
Schematic of the tandem foam extrusion line.

For bead foaming with 99.5 pure CO_2_ (Rießner Gase), a) twin-extrusion line (Reifenhäuser, Troisdorf, Germany) (Figure 1) was used. The setup was a co-rotating twin screw extruder with an overall length of 1857.5 mm (cylinder), inner diameter of 42.85 mm (screw diameter 42.3 mm), combined with a single screw cooling extruder equipped (1346 mm long and 34 mm screw core diameter). The zone temperatures in the A-extruder were adjusted to 140–260 °C with a pressure of 300 bar and in the B-extruder to 180–260 °C with an average pressure around 120 bar. The bead foaming mechanism follows the well-known procedure for foam extrusion/bead foaming with a tandem setup, which was also discussed and described in a review article by two of the authors [8,27]. First, the polymer was molten in the dosing area of a twin-screw extruder, mixed with the blowing agent and homogenized to a single-phase solution before reaching the B-extruder. In this single-screw extruder, the homogeneous melt-gas-solution is usually cooled to a lower temperature to counter the reduction in viscosity by dissolution of the blowing agent [28,29] and finally conveyed to the gear pump (controls the pressure before the die) and then to the die plate (multiple holes) while increasing the pressure toward the die. After the melt leaves the die, it begins to expand and is subsequently separated into beads by rotating knifes, cooled down through circulating water. The final beads are dried and ejected by the underwater pelletizer.

The steam chest molding of the beads was performed by the NMB GmbH with a high-pressure Steam-Chest-Molding (SCM) Machine TVZ162/100PP (Teubert Machienenbau GmbH, Germany) for EPET and EPC parts with a density of 200 ± 10 kg/m^3^ and a geometry of 300 × 200 × 15 mm^3^. The used pressures were in the range of 12–14 bar for EPC and around 20 bar for EPET. EPP was welded with a Transtec72/52PP SCM (Teubert Machienenbau GmbH, Germany) with the same geometry and density ranges, but for welding EPP, a lower pressure around of 4–5 bar was sufficient.

### 2.3. Material Characterization

Scanning electron microscopy (SEM) measurements were carried out on graphite-sputtered samples with a scanning electron microscope (SEM, Model: JEOL JSM-6510, Borken, Germany) at 1.5 kV and evaluated with ImageJ according to the “circle of equal projection area” method. Dynamic-mechanical analysis (DMA) was done using a DMA (Model: Gabo Eplexor 500N, NETZSCH GmbH, Selb, Germany) in compression (15 × 15 × 15 mm^3^ specimen) with temperatures ranging from −25 to 250 °C, a heating rate of 1 K/min, a strain-controlled mode with an amplitude of 5% (static) and 2% (dynamic) sample height with a frequency of 1 Hz. For further characterization of the final parts, compression tests and three-point bending were performed at room temperature, 80 °C, and 110 °C. Additionally, impact tests were conducted at room temperature (RT) (falling dart). Compression testing was performed according to DIN EN ISO 844 with a universal testing machine (Zwick Z020, Zwick & Roell, Ulm, Germany) with a specimen geometry of 15 × 15 × 15 mm^3^, and a 10 kN load cell up to 60 % compression. Three-point bending was carried out based on ISO 1209-1 with the same testing machine, 10 mm/min, and a 20 kN load cell. Specimens were skinned to suppress the influence of the outer layer, and the geometry was modified to 120 × 25 × 10 mm^3^. Tensile properties were studied according to the DIN EN ISO 53430 on a universal testing machine (Zwick Z050, Zwick & Roell, Ulm, Germany) with a specimen geometry of 140 (30+80+30) × 80 × 15 mm^3^ with a load cell of 20 kN. Impact was tested on a Falling Impact Tester (Fractovis Plus, Instron Ceast, Pianezza, Italy) with a max. energy of 40 J and an impact velocity of 4.4 m/s (based on DIN EN ISO 6603-2) on a 60 × 60 × 15 mm^3^ specimen. All mechanical experiments were performed at room temperature, 80, and 110 °C with a 10 min step for temperature equilibrium prior to testing the specimen of the same density of 200 kg/m^3^. Thermal conductivity measurements were carried out with a black-box device (HFM 446 Lambda, NETZSCH, Selb, Germany) at –10, 10, 25, 50, and 70 °C.

## 3. Results

### 3.1. Foamed Beads and Foamed-Part Morphology

**Figure 2:**
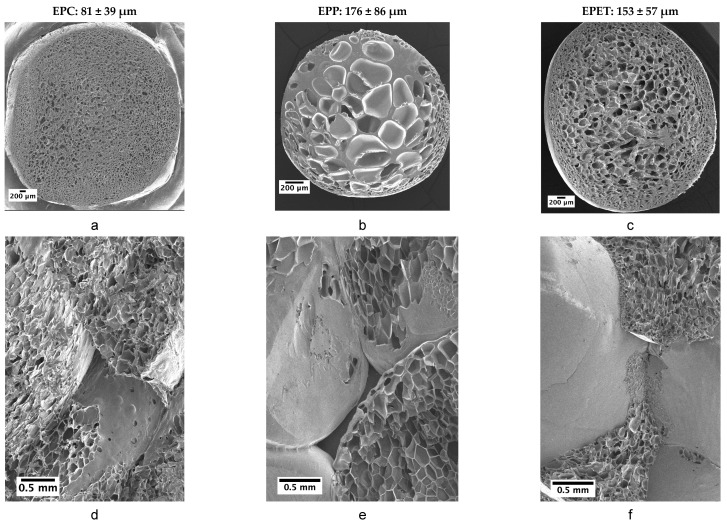
SEM pictures of the foamed beads of (**a**) expanded PC (EPC), expanded PP (**b**) (EPP), and expanded PET (**c**) (EPET) and corresponding parts EPC (**d**), EPP (**e**) and EPET (**f**) at 200 kg/m^3^.

EPP is one of the most used bead foams on the market, mostly due to its easy production and the well-established molding of parts. EPP is usually produced in discontinuous batch foaming processes, which leads to a double melting peak due to annealing. Nevertheless, the morphology in the bead foam is inhomogeneous with a high average cell diameter of 176 ± 86 µm, which is reduced during molding (Figure 2b). For welding, simple aluminum molds, as well as low steam pressures up to 6 bars, suffice, which is one of the advantages of this material. The SEM picture of the welded EPP part shows clear molding borders and a few inter-bead fractures (Figure 2e). 

In comparison to that, EPC and EPET are produced in a continuous bead-foam extrusion. EPET exhibits larger cells in the core and a smaller cells near the outer shell with an average cell diameter of 153 ± 57 µm and improved morphology compared to EPP (Figure 2c), compared to the EPP bead. The molded part shows a few well-fused areas, but significantly more inter-bead ruptures than EPP, which should correspond to a worse welding quality and, thus, inferior mechanics as well (Figure 2f). The reason for that is the high melting point of PET, which can only be reached by welding in a high-pressure machine and mold with pressures up to 20 bars. The continuous manufacturing process for EPC with CO_2_ has been developed and improved, compared to existing literature, to such an extent that round beads with a density of around 200 kg/m^3^ (~400 kg/m^3^ in comparable work from literature [19]) can be realized. Despite having the largest beads of all three materials, EPC has the most homogeneous morphology with an average cell diameter of 81 ± 39 µm (Figure 2a). Furthermore, EPC has to be welded with the high-pressure SCM machine as well, to reach sufficient temperatures for bead-fusion. Comparing the SEM pictures of the molded parts, EPC shows fewer inter-bead ruptures and a good fusion quality between the beads (Figure 2d). 

In general, the morphology of the final parts resembles the bead foam with slight compression or additional expansion of the original cells. It is known and reported in the literature that smaller and homogeneous distributed foam cells result in better mechanical performance [30]. Therefore, the mechanical properties depend mainly on the foam morphology and the bead size, but also on foam density and base polymer.

Additionally, the influence of the molecular structure on the rheological and mechanical properties should be discussed. According to the literature for the polyesters molecular weight, molecular weight distribution, as well as branching/crosslinking, affect the melt strength. Higher melt strength prevents cell coalescence during foaming and results in a higher density reduction, as well as a more homogeneous and closed cell distribution. Based on that, a more homogeneous foam morphology results in higher mechanical performance (e.g., higher compression strength) [31,32,33,34]. Furthermore, according to the work of Engelberg et al., a higher molecular weight (5-fold) enables an increase in tensile strength up to 20%, which strongly depends on the polymer type. In addition, a threshold value is expected with no further influence on mechanics [35]. As for the material used in this study, it is hard to tell the composition for the commercially available systems. Additionally, the influence of the molecular structure on the mechanical performance is still ongoing research and will be reported in future articles on E-PC.

### 3.2. Temperature-Dependent Mechanical Properties 

#### 3.2.1. Dynamic-Mechanical Analysis (DMA) of EPC in Comparison to EPP and EPET

**Figure 3:**
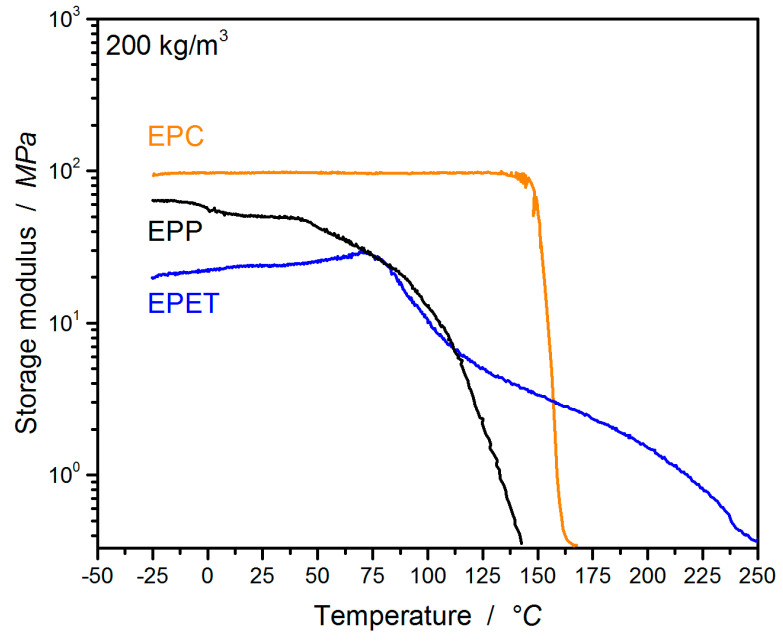
Dynamic-mechanical analysis (DMA) temperature sweep (1 K/min) for EPP, EPC, and EPET bead foam parts at 200 kg/m^3^.

DMA temperature sweeps in compression were performed for EPP, EPC, and EPET bead foam parts of 200 ± 10 kg/m^3^ for comparability and to determine the temperature-dependent modulus, as well as the glass transition temperature (T_g_) of the parts. In Figure 3, the storage moduli as a function of the temperature are plotted. EPC proves to be temperature-insensitive and retains a high modulus of about 100 MPa over a broad temperature range until 135 °C. Close to the T_g_, the storage modulus begins to decrease. Up to its T_g_ around 70 °C, EPET displays an increasing modulus, possibly due to recrystallization. At –25 °C, EPC has a modulus of 96 MPa, which is 1.5 times higher compared to EPP (64 MPa) and almost five times higher than EPET (22 MPa). At 25 °C, the storage modulus of EPC is 50% higher compared to EPP (50 MPa) and 75% higher than EPET (23 MPa). At elevated temperatures of 80 °C, the EPC modulus is increased by 400% compared to EPP (25 MPa) and by 500% compared to EPET (20 MPa). At the highest measuring temperature for the other experiments in this study (110 °C), EPC shows a 12 times higher performance regarding the storage modulus, compared to EPP (8 MPa) and EPET. The overall low modulus of EPET surpasses EPP only above 115 °C and EPC above 158 °C. The DMA analysis confirms very well the potential of EPC for higher-temperature applications, while other bead foams like EPP and EPET exhibit a distinct temperature dependency of their modulus above their glass transition temperature, as well as even a lower modulus compared to EPC below their T_g_.

#### 3.2.2. Compression Properties of EPC in Comparison to EPP and EPET

**Figure 4:**
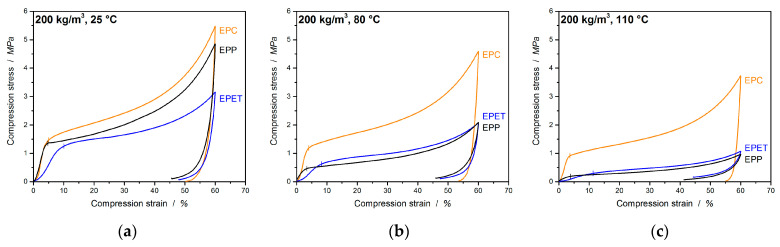
Stress–strain curves under compressive loading of EPC, EPP, and EPET at 25 (**a**), 80 (**b**), and 110 °C (**c**).

In many cases, polymer foams are subjected to a compressive loading; therefore, it is important to characterize the mechanical performance under this condition. EPC is a bead foam for high-temperature applications where commodity polymers like EPP fail. There are also some competing materials in this field of application, such as EPET, which are also used for direct comparison at the same density in Figure 4. This already visualizes the higher compressive performance of EPC compared to EPP and EPET. 

**Figure 5:**
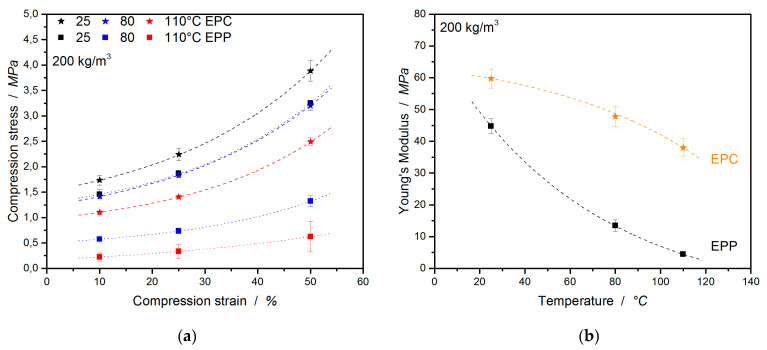
Comparison of compression strength (**a**) and compression moduli (**b**) of EPC and EPP at 25, 80, and 110 °C.

In Figure 5a, EPC, EPP, and EPET show similar behavior, with a compressive strength (at 10% strain and 25 °C) of 1.73 MPa for EPC, 1.46 MPa for EPP, and 1.25 MPa for EPET. At 80 °C and above, EPC is clearly less temperature-sensitive (also the resilience) and, hence, superior in compression performance compared to the other foams. EPC retains a compressive strength of 1.42 MPa, 60% higher compared to EPP (0.57 MPa) and 50% higher compared to EPET (0.67 MPa). An increase in the temperature to 110 °C leads to a 35% drop in the compressive strength of EPC (1.1 MPa). Nevertheless, the EPC final part still shows a 78% higher performance compared to EPP (0.24 MPa) and EPET (0.24 MPa). The difference in performance is evident, with the compressive strength of EPP at room temperature being just as good as the performance of EPC at 80 °C. 

The compressive moduli follow a similar trend (Figure 5b). EPC’s compression modulus is 25% higher (59.7 MPa) than the value of EPP at 25 °C, 72% higher at 80 °C, and 88% higher at 110 °C. 

**Figure 6:**
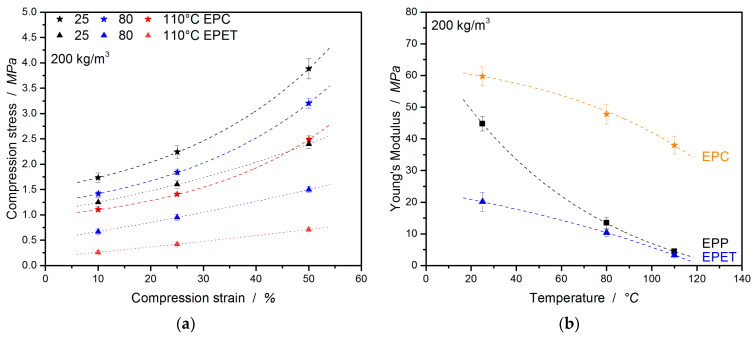
Comparison of compression strength (**a**) and compression moduli (**b**) of EPC to EPET at 25, 80, and 110 °C.

In the further course of this study, the compressive properties of EPC and EPET will be compared. Already at room temperature (full data set in Table 2), EPC expresses significantly higher mechanical properties than EPET (same density) as shown in Figure 6a,b. With a compressive strength of 1.73 MPa (at 10 % strain), EPC performs 27% better compared to EPET (1.25 MPa at 10%). Comparing the compression properties at 80 and 110 °C shows that EPC outperforms EPET by a factor of 2 (at 80 °C) and by a factor 4 (at 110 °C). Furthermore, EPET has the lowest measured compression modulus in this comparison. EPC has a 68% higher compression modulus at RT compared to EPET, 79% higher at 80 °C, and 92% higher values at 110 °C. The drop in mechanical properties of EPET is caused by the glass transition around 70 °C. It therefore shows the worst compression performance and has even lower or equal compressive characteristic properties at elevated temperatures as EPP. This means that EPC is superior at all tested temperatures in terms of mechanical performance under pressure compared to EPP and EPET. The full data set for this comparison is summarized in Table 2.

#### 3.2.3. Tensile Properties of EPC in Comparison to EPP and EPET

**Figure 7:**
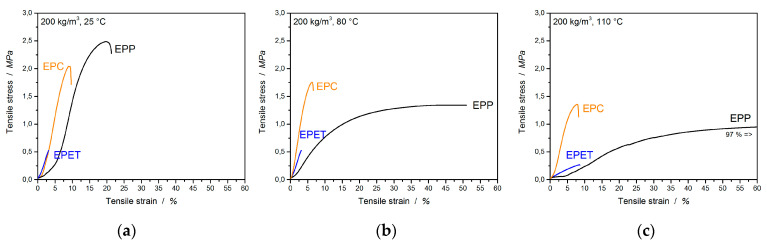
Tensile curves for EPC, EPP, and EPET at 25 (**a**), 80 (**b**), and 110 °C (**c**).

Tensile tests were carried out in order to obtain the tensile properties, as well as tendencies of the welding quality. In Figure 7, the tensile curves for EPP, EPC, and EPET are displayed at different temperatures. The most apparent observation is the temperature-induced ductility of EPP ranges from 20% (25 °C) up to 100% (110 °C) in Figure 7a–c. 

**Figure 8:**
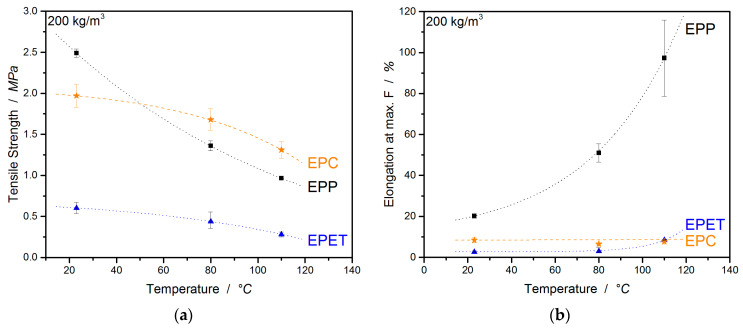
Tensile strength (**a**) and elongation at max Force (**b**) of EPC, EPP, and EPET at 25, 80, and 110 °C.

For better visualization of the characteristic values, they are displayed as a function of temperature. Figure 8a,b show a comparison of the tensile strength and elongation at break of EPP, EPET, and EPC in a direct comparison at the same density of 200 kg/m^3^. When considering the tensile strength, EPP shows the highest values at room temperature of 2.49 MPa, followed by EPC with 1.97 MPa and EPET with the lowest value of 0.60 MPa. With increasing temperatures, all compared materials lose tensile strength. For example, EPP loses 45% of its tensile strength at 80 °C and 61% at 110 °C. EPC, on the other hand, can provide significantly better tensile properties at elevated temperatures due to the high T_g_ of 150 °C. The polycarbonate bead foam has the highest tensile strength in this comparison with 1.68 and 1.31 MPa (at 80 °C and 110 °C respectively). 

The second important value for bead foams resulting from tensile experiments is the elongation at break. In the literature, elongation at break or elongation at max. force is often reported to represent tendencies of the welding quality of the bead foam at room temperature. 

At RT, EPP shows an elongation at max. F of 20%, a very high value that represents good welding quality. At a temperature of 80 °C, the value rises to 51 % and, at 110 °C, increases even further to 97%. PP is a semi-crystalline material with a T_g_ around 0 °C and a T_m_ around 140 °C. This means that EPP foams are usually above T_g_ and behave significantly more plastic at those temperatures (creep behavior) than other materials below the glass transition. EPET exhibits an *elongation at max. F* of only 2.7% at RT and takes a maximum value of 8.4% at 110 °C with the temperature increase. This shows that the welding quality of EPET is not as good as EPP (20%) and EPC (8.4%). Here too, the glass transition is exceeded due to the temperature increase, so the material becomes significantly more plastic, which is why the *elongation at max. F* increases. As expected, EPC shows significantly lower temperature sensitivity and performs relatively consistently in regard to the *elongation at max. F* (Table 3). It varies from 8.4% (RT) to a value of 7.7% (110 °C) and remains at a similar value when the standard deviation is considered. 

**Figure 9:**
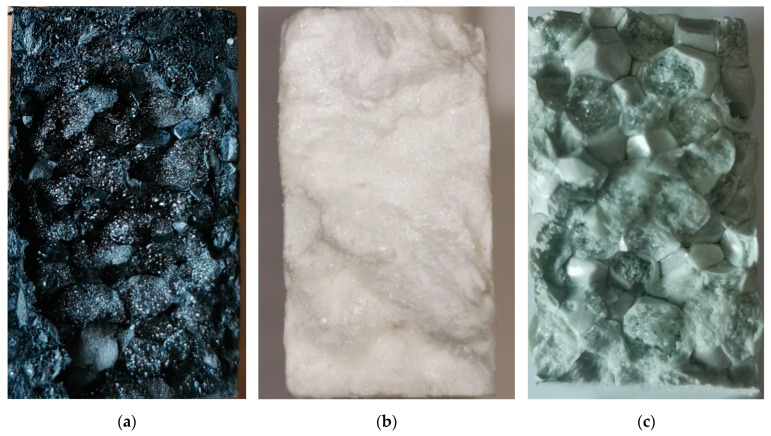
Fracture surfaces of the bead foam parts in tensile load for (**a**) EPP, (**b**) EPC, and (**c**) EPET.

Additionally, the fractured surfaces of the tensile specimen are considered in Figure 9 to visualize the welding qualities of the bead-foamed parts. In Figure 9a, the fracture surface of EPP shows mainly intra-bead rupture, as well as a few areas where inter-bead rupture occurs. EPC (Figure 9b), on the other hand, despite having lower *elongation at max*. F compared to EPP, showed almost only intra-bead rupture, confirming the good fusion quality of the foam. In conclusion, the bad welding quality of EPET can be confirmed by the fracture surface of the specimen, as a lot of inter-bead rupture occurred (Figure 9c).

EPC is a continuously produced foam with good fusion quality (amorphous). EPET is a continuously produced semicrystalline (challenging welding) bead foam. The foaming window is narrow due to its semi-crystalline nature [29], as well as the welding window. Additionally, a very high steam pressure (~20 bar) is required to reach the welding temperature around 260 °C (EPET). EPP, on the other hand, can be molded easily (even though it is semicrystalline) due to the double melting peak usually generated by the discontinuous batch foaming. Additional advantages are low steam pressures of 4–6 bar and aluminum molds being sufficient to fuse EPP beads.

#### 3.2.4. Bending Properties of EPC in Comparison to EPP and EPET

**Figure 10:**
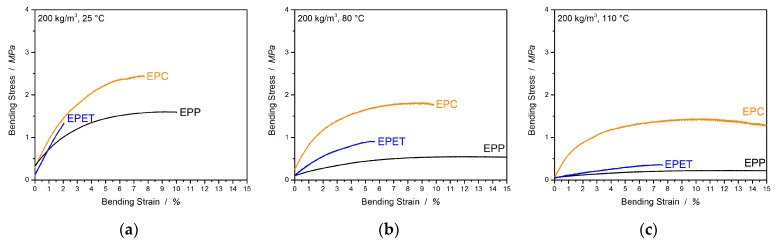
Three-point-bending curves for EPC, EPP, and EPET at 25 (**a**), 80 (**b**), and 110 °C (**c**).

**Figure 11:**
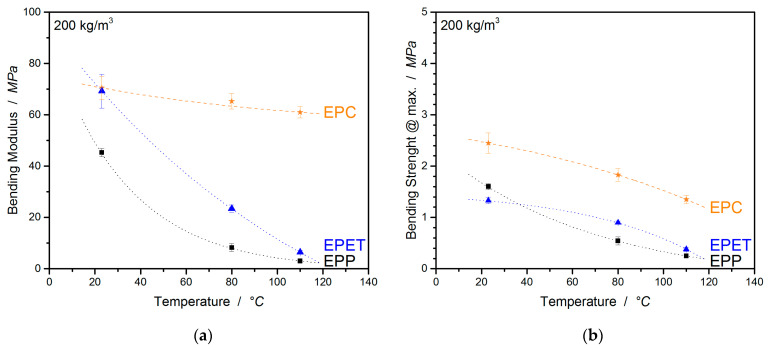
Temperature-dependent bending modulus (**a**) and strength (**b**) of EPC, EPP, and EPET.

Another relevant type of load for polymer foams is bending. Here, the final part experiences complex loads by subjecting the upper side to a compressive load and the underside to tension. This enables a statement about the bending stiffness of the part and about the welding quality, which is represented by the maximum bending strain. In addition to that, the values of the elongation at break from tensile tests are compared with the bending elongation values of the three-point bending tests. The preparative effort for the tensile test specimens is relatively high and the information gained for foams is of a lesser value, as foams are usually subjected to pressure and bending loads. In the “high”-temperature application field, EPC has to compete with the usual standard EPP, as well as EPET. Therefore, in Figure 10, the raw three-point bending curves are shown, which illustrate the temperature-induced creeping behavior (especially for EPP as the setup maximum is set to 15% and EPP maxes it at 80 °C), as well as the bending moduli, and the strength of EPC, EPP, and EPET is directly compared in Figure 11a–c. 

At room temperature, EPC has the highest flexural modulus of 70.5 MPa followed by EPET with 69.2 MPa. EPP, on the other hand, performs comparatively poorly at a density of 200 kg/m^3^ with 45.3 MPa. As soon as bending properties at elevated temperatures are considered (80 °C), the modulus for EPP drops by 81.5%, and for EPET, by 65.4%. EPC, on the other hand, has a continuous service temperature of up to ~130 °C and only exhibits a modulus drop of 7.4% (65.3 MPa) at a temperature of 80 °C, and has, by far, the highest modulus of all considered materials in this comparison. The same trend continues at 110 °C. EPC has a 20 × higher bending modulus than EPP and 10 × higher than EPET. In terms of flexural strength, EPC is superior to other materials at all tested temperatures. EPP and EPET clearly lose rigidity with increasing temperature and have a low flexural strength around 0.2–0.4 MPa (110 °C), while EPC is 3–4 times stronger and represents the good bending performance of EPC at elevated temperatures.

The welding quality is quantified based on the maximum bending strain and is compared in Table 4, as the *bending at break* sometimes has to be manually adjusted in the software, making it uncertain. As, at higher temperatures, some of the materials start to creep significantly, only the values at 25 °C are used for the discussion of welding quality. In this comparison, EPP shows the best bead fusion with a *bending strain at max. F* of 9.6% followed by EPC with 7.7% and the worst welded material EPET with a bending strain of only 1.9%. The comparison of the *elongation at max. F* with the *bending strain at max.* in Table 4 shows that the three-point bending test generates much more moderate strains based on the experimental procedure than tensile results. EPP shows a maximal bending strain of 9.6%/11/13% at 25, 80, and 110 °C respectively, while the values of *elongation at max. F* are significantly higher with 20.2/51.0/97.3% at 25/80/110 °C, implying that the data from the three-point-bending test are more reliable at RT and especially at higher temperatures due to a reduced creep-sensitivity of the testing setup. Further, EPET exhibits a *bending strain at max F.* of only 1.9% at RT and takes a maximum value of 7.6% at 110 °C with the temperature increase. Similar tendencies with comparable values (2.7–8.4%) are also generated in the tensile experiments. This confirms the worse welding quality of EPET compared to EPP and EPC, as well as the reliability/comparability of the measured data. For EPC, the *bending strain at max. F* varies from 7.7% (RT) to a value of 9.5% (110 °C) and shows comparable values to the tensile experiments (8.4/6.6/7.7) in regard to the standard deviation. The values for *elongation at max. F* are in good agreement (less than 10% standard deviation) with the values for the *bending strain at max*. *F* and enable the same statement about fusion quality. Therefore, it can be concluded that the three-point bending tests can provide more reliable data on the welding quality, with less deviation and effort, than the tensile experiments.

#### 3.2.5. Temperature-Dependent Impact-Behavior of EPC Compared to EPP and EPET

**Figure 12:**
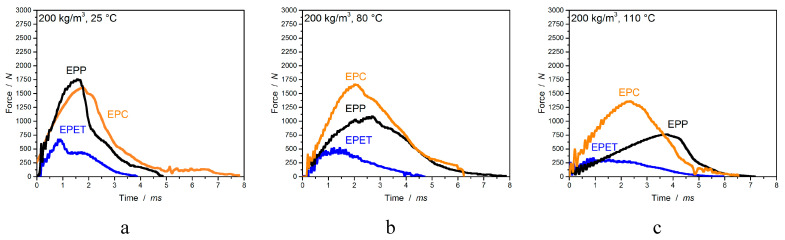
Impact diagrams for EPP, EPC, and EPET at 25 (**a**), 80 (**b**), and 110 °C (**c**) with a velocity of 4.4 m/s.

The last mechanical characterization discusses the material impact performance at different temperatures. This investigation allows statements about the force required for penetrating the specimen and the amount of absorbed energy. The last is represented by the area enclosed beneath the corresponding curve displayed in Figure 12. Additionally, the figure visualizes the temperature-induced drop in absorbed energy and a plasticized deformation at higher temperatures for EPP (max. peak shift). 

**Figure 13:**
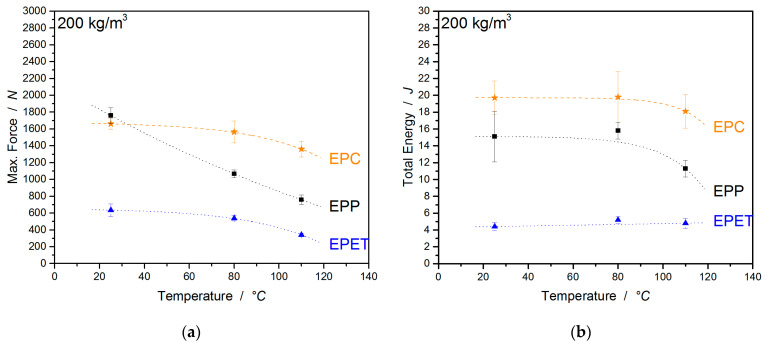
Comparison of impact force (**a**) and energy absorption (**b**) of EPC, EPP, and EPET atRT, 80, and 110 °C.

Further, EPC is compared to EPP and EPET in a more detailed display in Figure 13a,b. At room temperature, EPP expresses the highest required force of 1760 ± 94 N for penetration, followed by EPC with 1661 ± 66 N and with a low value of 635 ± 76 N for EPET. Welding quality plays an important role for impact performance and accordingly influences the required penetration force. For example, EPP and EPC show high values for impact resistance, while EPET (poor welding quality) requires almost 1/3 less force for penetration. Above approx. 35 °C, EPC has a higher impact resistance than EPP. At temperatures of 80 °C, EPC has a 500 N higher resistance to penetration (1565 ± 130 N) than EPP. At 110 °C, the values drop only by 18% for EPC, while EPP already lost 57% of its impact resistance. Despite the overall lower values for EPET, the temperature-induced decrease of 46% in the characteristic values at 110 °C is lower than that for EPP. 

However, if the absorbed energy of the materials is considered, EPC expresses with 19.7 J a significantly higher energy absorption than EPP with 15.1 J and especially EPET with 4.4 J. The amount of absorbed energy can be considered steady for all materials up to 80 °C in regard to the standard deviation. At a measuring temperature of 110 °C, however, the absorbed energy falls with almost all materials. EPC loses 8% of the characteristic value, EPP loses 25%, while EPET shows a further increase in the absorbed energy. The decrease is caused by higher polymer chain mobility and reduced stiffness, while for EPET, the annealing step improves the poor welding quality. The exact data and the deformations for this comparison can be found in Table 5.

#### 3.2.6. Temperature-Dependent Thermal Conductivity

**Figure 14:**
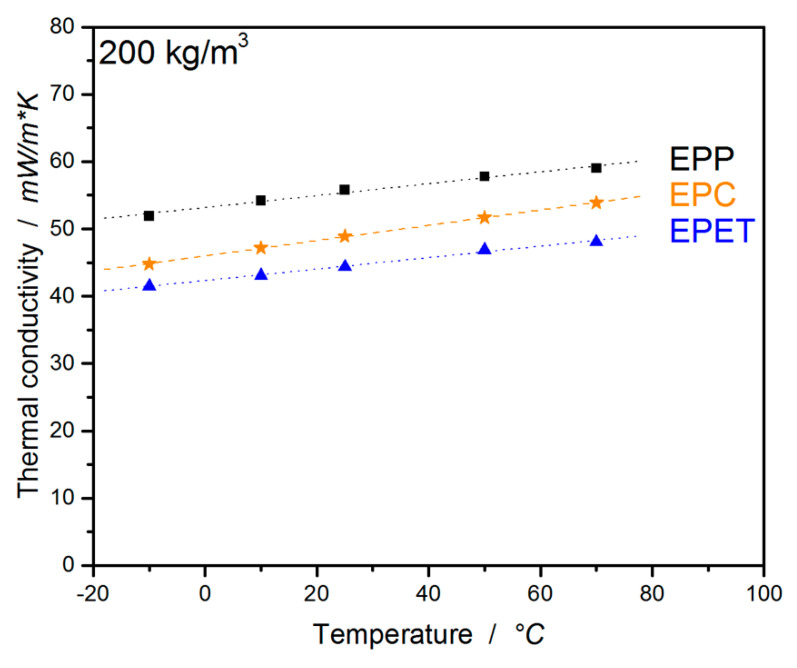
Temperature-dependent thermal conductivity of EPC, EPP, and EPET.

Another interesting property for foams is the insulating capability in the form of a very low thermal conductivity. This material property makes foams predestined for insulating applications. Thus, EPC, EPP, and EPET were characterized for their thermal conductivity as a function of temperature (ranging from −10 to 70 °C). As depicted in Figure 14, thermal conductivity shows a linear behavior and dependence on temperature and is mainly influenced by density and morphology. Usually, the material supplier provides a thermal conductivity at 10 °C for their data sheets; thus, this value will be used for comparison. EPP shows a comparatively high value of 54 mW/m*K at a density of 200 kg/m^3^, but is usually used at a significantly lower density (also lower thermal conductivity). On the contrary, polycarbonate as raw material already has a low thermal conductivity of 200 mW/m*K, so the corresponding foam with a low value of 47.2 mW/m*K stands out. The lowest thermal conductivity of 43.1 mW/m*K, by far, in this comparison is exhibited by EPET at 10 °C, due to small beads, even though they are not as well welded as the other two materials. In addition, EPP and EPET are obviously nucleated (colored beads), which enables smaller and finer cell morphology and, thus, lower thermal conductivities. EPC, on the other hand, is not.

If the density dependency is taken into account, the thermal conductivity can still be optimized to lower values by reducing EPC density or using nucleating agents to optimize morphology. The complete dataset can be found in Table 6.

## 4. Conclusion

Within this work, a new polymer bead foam for high-temperature applications was introduced. The thermo-mechanical performance of EPC in compression, bending, tensile, impact, and the insulating properties was studied and compared to the established EPP and EPET at a final part density of 200 kg/m^3^ and best achievable welding quality. EPP and EPC showed a good welding quality with less voids, which was represented by mainly intra-bead rupture occurring in the fractured surface pictures. The first generation of EPET, on the other hand, requires extreme pressures for welding, and the resulting overall welding quality was poor at a density of 200 kg/m^3^, which was addressed in further material development. Nevertheless, the generated mechanical key-values for EPET correlate well with the product brochure for ArmaShape [7].

**Figure 15:**
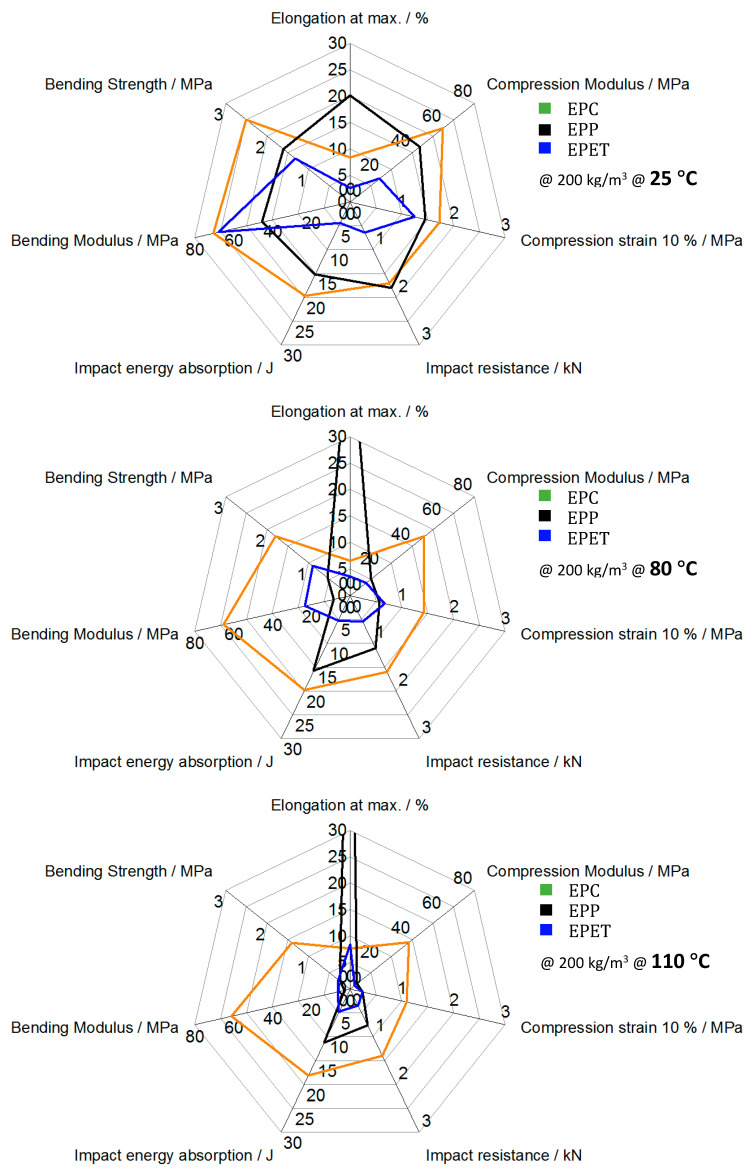
Graphical conclusion of temperature-dependent mechanics of EPC, EPP, and EPET.

The DMA trials showed thermo-mechanical properties of the foams with comparable behavior and transitions to the neat polymers, respectively. Above 80 °C, the advantages of EPC become more visible. While the other foams suffer from a decreasing modulus over a temperature range from 25 to 100 °C, EPC expresses a significantly reduced temperature sensitivity up to 135 °C. It can be concluded that EPC has excellent properties at RT and outstanding mechanical performance at temperatures above 100 °C in regard to bending, compression, and impact in the current development state, as shown in the graphical conclusion in Figure 15. EPP, a well-established material, on the other hand, loses its mechanical performance around 80 °C and starts to deform and to creep irreversibly. Thus, this research shows the potential and advantages of EPC compared to other established foams. The application fields for this kind of mechanical performance can range from protective clothing with constant mechanical properties in winter, as well as in summer, near-engine applications in the automotive sector, as well as battery covers with insulating properties in electric cars. 

## Figures and Tables

**Table 1:** Literature review for polycarbonate foams.

Author	Material	Setup	Parameter	Results
Kumar et al. [14] (1994)	Lexan ^®^ 9030	Batch	Gas-loading: 22 °C, 50 bar Loading-time: 70 h Foaming: 60−160 °C	Max. uptake: 9 wt.% Cell densities: 1–10 × 10^9^/cm^3^ Foam densities: 0.1–1.2 g/cm^3^
Ma et al. [16](2013)	not defined	Batch	Gas-loading: 60 °C, 100–200 bar Loading-time: 6 h Foaming: 110–160 °C Foaming-time: 5–30 s	Max. uptake: 8–11 wt.% Cell densities: ~1.39 × 10^9^/cm^3^ Foam densities: 0.05–0.35 g/cm^3^ Cell diameter: 7.2–10.7 μm
Ma et al. [16](2013)	not defined	Batch	Gas-loading: 60 °C, 200 bar Loading-time: 6 h Foaming: 150 °C, 10 s Depression: 100–140 bar, Foaming: 10 s	Max. uptake: 11 wt.% Foam densities: 0.45 g/cm^3^ Cell diameter: 1–50 μm Tensile strength: 35 MPa E-modulus: 400 MPa
Kumar et al. [22] (1994)	Lexan ^®^ 9030	Batch	Gas-loading: 20 °C, 50 bar Loading-time: 6 h Foaming: 110 °C Foaming-time: 30 s	Compact layer: 0–250 μm
Bao et al. [23] (2013)	PLA/PC blend (Nature-Works ^®^ 2002D/K-1300)	Batch	Gas-loading: 110 °C, 210 bar Loading-time: 2 h Foaming: 110 °C Foaming-time:10 s	PC-phase Cell densities: 3.30 × 10^14^/cm^3^ Cell diameter: 45.25 nm PLA-Phase Cell densities: 6.26 × 10^8^/cm^3^ Cell diameter: 6.36 μm
Kumar et al. [20] (1994)	Lexan ^®^ 9030	Batch	Gas-loading: 22 °C, 55 bar Loading-time: 60 h Foaming: 60−180 °C Foaming-time 30 s Desorption: 30 min	Cell densities: 2.40–6.37 × 10^9^/cm^3^ Cell diameter: 1.6–8.9 μm Tensile strength: 3–45 MPa E-modulus: 64–1780 MPa
Weller et al. [21] (2010)	Lexan ^®^ 9030	Batch	Gas-loading: 27 °C, 10–60 bar Loading-time: 60 h Foaming: 105–139 °C Foaming-time: 3 min Desorption: 5 min	Foam density: 0.56 g/cm^3^ Cell densities: 2.6–37.1 μm Tensile strength: 25–29 MPa E-modulus: 836–978 MPa
Bureau et al. [24] (2006)	Lexan ^®^ GE 9034	Batch	Gas-loading: 22 °C; 4, 5 bar Loading-time: 10, 14 days Foaming: 86, 89 °C Foaming-time: 10 min	Densities: 0.7, 0.9 g/cm^3^ Cell diameter: 10 μm Cell densities: ≈ 10^9^/cm^3^ Strain at break: 0.57, 0.80 mm/mm
Seeler et al. [25] (1994)	Lexan ^®^ 9030	Batch	Gas-loading: 22 °C, 7−55 bar Loading-time: 60 h	Yield strength: 50–65 MPa E-modulus: 2300–2500 MPa
Mascia et al. [26] (2006)	Paltuf™ ^®^	Batch	Gas-loading: 180 °C, 7–55 bar	ΔH_f_ = 26.7 J/g Crystallinity: 24 %
Park et al. [18] (2005)	Lexan ^®^ 101–112	Extrus.	BA-conc.: 3, 5, 7 wt.% CO_2_ Die-temperatures: 150–240 °C	Foam densities: 0.085 g/cm^3^
Gendron et al. [19] (2003)	Lexan ^®^ 101 K-2870	Extrus.	BA-conc.: 0–3.0 wt-% CO_2_ 0–8 wt.% n-Pentane	Foam densities: 0.4–0.7 g/cm^3^ Cell diameter: 2–5 μm

**Table 2:** Compressive key values for the comparison of EPC, EPP, and EPET.

		**EPC**	**EPP**	**EPET**
**25 °C**	**Young‘s Modulus/MPa**	59.7 ± 3.0	44.8 ± 2.3	18.9 ± 2.0
	**Stress at 10% strain/MPa**	1.73 ± 0.10	1.46 ± 0.09	1.25 ± 0.9
	**Collapse stress/MPa**	1.5 ± 0.2	1.4 ± 0.2	1.3 ± 0.2
	**Work until max. F/J**	5.2 ± 0.2	4.9 ± 0.2	3.5 ± 0.2
		**EPC**	**EPP**	**EPET**
**80 °C**	**Young‘s Modulus/MPa**	47.8 ± 3.1	13.5 ± 1.8	10.4 ± 1.1
	**Stress at 10% strain/MPa**	1.42 ± 0.05	0.57 ± 0.04	0.67 ± 0.5
	**Collapse stress/MPa**	1.2 ± 0.2	0.5 ± 0.2	0.6 ± 0.2
	**Work until max. F/J**	4.2 ± 0.1	2.0 ± 0.1	2.1 ± 0.1
		**EPC**	**EPP**	**EPET**
**110 °C**	**Young‘s Modulus/MPa**	38.0 ± 2.8	4.5 ± 0.2	2.8 ± 0.6
	**Stress at 10% strain/MPa**	1.10 ± 0.03	0.24 ± 0.08	0.24 ± 0.4
	**Collapse stress/MPa**	0.9 ± 0.2	0.2 ± 0.2	0.3 ± 0.2
	**Work until max. F/J**	3.4 ± 0.1	0.9 ± 0.1	1.0 ± 0.1

**Table 3:** Comparison of *elongation at max. F* and *tensile strength* of EPC, EPP, and EPET.

		**EPC**	**EPP**	**EPET**
**25 °C**	**Tensile strength/MPa**	1.97 ± 0.14	2.49 ± 0.05	0.60 ± 0.07
	**Elongation at max F./%**	8.4 ± 1.2	20.2 ± 1.1	2.7 ± 0.4
		**EPC**	**EPP**	**EPET**
**80 °C**	**Tensile strength/MPa**	1.68 ± 0.13	1.36 ± 0.06	0.46 ± 0.10
	**Elongation at max F./%**	6.6 ± 0.5	51.0 ± 4.5	3.6 ± 1.1
		**EPC**	**EPP**	**EPET**
**110 °C**	**Tensile strength/MPa**	1.31 ± 0.10	0.97 ± 0.02	0.28 ± 0.03
	**Elongation at max F./%**	7.7 ± 0.6	97.3 ± 18.6	8.4 ± 0.3

**Table 4:** Summary of bending characteristic-values of EPC, EPP, and EPET at 25, 80, and 110 °C.

		**EPC**	**EPP**	**EPET**
**25 °C**	**Bending-Modulus/MPa**	70.5 ± 4.5	45.3 ± 1.6	69.2 ± 6.6
	**Bending-Strength/MPa**	2.5 ± 0.20	1.60 ± 0.06	1.32 ± 0.05
	**Bending strain at max. F/%**	7.7 ± 1.3	9.6 ± 0.7	2.2 ± 0.2
	**Elongation at max. F (Tensile)/%**	8.4 ± 1.2	20.2 ± 1.1	2.7 ± 0.4
		**EPC**	**EPP**	**EPET**
**80 °C**	**Bending-Modulus/MPa**	65.3 ± 3.0	8.3 ± 1.5	23.4 ± 1.5
	**Bending-Strength/MPa**	1.8 ± 0.12	0.54 ± 0.8	0.90 ± 0.03
	**Bending strain at max. F/%**	8.0 ± 1.0	11.0 ± 0.5	5.5 ± 0.8
	**Elongation at max. F (Tensile)/%**	6.6 ± 0.5	51.0 ± 4.5	3.6 ± 1.1
		**EPC**	**EPP**	**EPET**
**110 °C**	**Bending-Modulus/MPa**	61.0 ± 2.2	3.0 ± 0.4	6.4 ± 0.8
	**Bending-Strength/MPa**	1.4 ± 0.10	0.25 ± 0.02	0.38 ± 0.04
	**Bending strain at max. F/%**	9.5 ± 0.5	13.0 ± 0.8	7.6 ± 0.8
	**Elongation at max. F (Tensile)/%**	7.7 ± 0.6	97.3 ± 18.6	8.4 ± 0.3

**Table 5:** Summary of impact characteristic values for EPC, EPP, and EPET at RT, 80, and 110 °C.

		**EPC**	**EPP**	**EPET**
**25 °C**	**Max. Force/N**	1661 ± 66	1760 ± 94	635 ± 76
	**Piercing Force/N**	829 ± 32	879 ± 49	315 ± 38
	**Total Energy/J**	19.7 ± 2	15.1 ± 3	4.4 ± 0.5
	**Total deformation/mm**	33.9 ± 8	20.4 ± 2	17.0 ± 1
		**EPC**	**EPP**	**EPET**
**80 °C**	**Max. Force/N**	1565 ± 130	1067 ± 46	543 ± 30
	**Piercing Force/N**	782 ± 65	553 ± 23	272 ± 15
	**Total Energy/J**	19.8 ± 3	15.8 ± 1	5.2 ± 0.4
	**Total deformation/mm**	26.4 ± 4	32.8 ± 1	19.8 ± 3
		**EPC**	**EPP**	**EPET**
**110 °C**	**Max. Force/N**	1361 ± 94	759 ± 55	341 ± 20
	**Piercing Force/N**	680 ± 47	379 ± 28	170 ± 10
	**Total Energy/J**	18.1 ± 2	11.3 ± 1	4.8 ± 0.6
	**Total deformation/mm**	30.0 ± 2	30.8 ± 2	26 ± 3

**Table 6:** Full data for thermal conductivity investigation for EPC, EPP, and EPET.

	Thermal Conductivity/mW/m*K					
	Neat polymer	−10 °C	10 °C	25 °C	50 °C	70 °C
**EPC 200 kg/m^3^**	200 ^#^	44.8	47.2	48.9	51.7	53.9
**EPP 200 kg/m^3^**	170–220 ^#^	51.9	54.2	55.8	57.8	59.0
**EPET 200 kg/m^3^**	240 *	41.5	43.1	44.4	46.9	48.1

^#^ Thermal conductivity according to material data sheet (PC by Covestro Deutschland AG, Germany; PP by Ineos Olefins & Polymers USA); * Material data sheet for bottle-grade PET; neat polymers refers to the neat matrix material of the bead foams.

## References

[1] Okolieocha C., Raps D., Subramaniam K., Altstädt V. (2015). Microcellular to nanocellular polymer foams: Progress (2004–2015) and future directions—A review. Eur. Polym. J..

[2] IFonseca I., Bräuer J., Graessel G., Hennenberger F., Birli R., Gutmann P. (2018). Advances in high performance thermoplastic foams Results and discussion. SPE Foams.

[3] Smith P.L.M., Hill J., Wardlaw A., Price D.S., Tarrier J. (2014). Expanded Polymer Pellets. U.S. Patent.

[4] (2012). Press Release, BASF SE, Neopolen P. Designed for New Ideas. www.basf.com/global/en/media/news-releases/2020/03/p-20-157.html.

[5] (1992). Product portfolio, BASF SE, Styropor - Technical Information. www.fepwaf33.basf.com/portal/basf/en/dt.jsp?setCursor=1_1222863&page=downloads.

[6] Standau T., Hädelt B., Schreier P., Altstädt V. (2018). Development of a Bead Foam from an Engineering Polymer with Addition of Chain Extender: Expanded Polybutylene Terephthalate. Ind. Eng. Chem. Res..

[7] (2018). Product portfolio, Armacell, ArmaShape (EPET) Product Brochure. https://local.armacell.com/en/armaform-pet-foam-cores/products/armashape/.

[8] Raps D., Hossieny N., Park C.B., Altstädt V. (2015). Past and present developments in polymer bead foams and bead foaming technology. Polymers (UK).

[9] (2019). Press Release, Asahi Kasei Europe GmbH, Polyamide Foam Product. www.asahi-kasei.eu/en/News/Premiere%20for%20PA%20Foam%20%E2%80%93%20Asahi%20Kasei%20at%20Foam%20Expo%20Europe%202019_n261.

[10] (2019). Press release, BASF SE, BASF entwickelt Ultramid® Partikelschaum für breites Anwendungsspektrum. www.basf.com/global/de/media/news-releases/2019/10/p-19-360.html.

[11] Dörr D., Raps D., Kirupanantham D., Holmes C., Altstädt V. (2020). Expanded polyamide 12 bead foams (ePA) thermo-mechanical properties of molded parts. AIP Conf. Proc..

[12] (2019). Asahi Kasei Europe GmbH, SunForceTM mPPE-Particle Foam Product Brochure. www.asahi-kasei.eu/en/News/SunForce™m-PPEParticleFoamAsahiKaseiintroducesnewlydevelopedlightweightmaterialforincreasedefficiencyandsafetyofelectricvehiclebatteries_n264.

[13] Hahn K., Hofmann M., Ruckdäschel H., Sandler J.k.W., Scherzer D. (2012). Particle foam based on a polymer including polystyrene, styrene copolymer, polysulfone or polyethersulfone, comprises inorganic filler e.g. talc having specified particle size and wax or oligomer based nucleating agent e.g. polyethylene wax. Patent.

[14] Kumar V., Weller J. (1994). Production of Microcellular Polycarbonate Using Carbon Dioxide for Bubble Nucleation. J. Eng. Ind..

[15] Holl M.R., Garbini J.L., Murray W.R., Kumar V. (2001). A steady-state mass balance model of the polycarbonate-CO_2_ system reveals a self-regulating cell growth mechanism in the solid-state microcellular process. J. Polym. Sci. Part B Polym. Phys..

[16] Ma Z., Zhang G., Yang Q., Shi X., Shi A. (2013). Fabrication of microcellular polycarbonate foams with unimodal or bimodal cell-size distributions using supercritical carbon dioxide as a blowing agent. J. Cell. Plast..

[17] Weller J.E., Kumar V. (2010). Solid-state microcellular polycarbonate foams. I. The steady-state process space using subcritical carbon dioxide. Polym. Eng. Sci..

[18] Lee J.W.S., Wang K., Park C.B. (2005). Challenge to Extrusion of Low-Density Microcellular Polycarbonate Foams Using Supercritical Carbon Dioxide. Ind. Eng. Chem. Res..

[19] Gendron R., Daigneault L.E. (2003). Continuous Extrusion of Microcellular Polycarbonate. Polym. Eng. Sci..

[20] Kumar V., VanderWel M., Weller J., Seeler K.A. (1994). Experimental Characterization of the Tensile Behavior of Microcellular Polycarbonate Foams. J. Eng. Mater. Technol..

[21] Weller J.E., Kumar V. (2010). Solid-state microcellular polycarbonate foams. II. The effect of cell size on tensile properties. Polym. Eng. Sci..

[22] Kumar V., Weller J.E. (1994). A model for the unfoamed skin on microcellular foams. Polym. Eng. Sci..

[23] Bao D., Liao X., He T., Yang Q., Li G. (2013). Preparation of nanocellular foams from polycarbonate/poly(lactic acid) blend by using supercritical carbon dioxide. J. Polym. Res..

[24] Bureau M.N., Kumar V. (2006). Fracture toughness of high density polycarbonate microcellular foams. J. Cell. Plast..

[25] Seeler K.A., Kumar V. (1994). Effect of CO_2_ Saturation and Desorption on the Fatigue Life of Polycarbonate. J. Eng. Mater. Technol..

[26] Mascia L., Re G.D., Ponti P.P., Bologna S., Giacomo G.D., Haworth B. (2006). Crystallization effects on autoclave foaming of polycarbonate using supercritical carbon dioxide. Adv. Polym. Technol..

[27] Köppl T., Raps D., Altstädt V. (2014). E-PBT - Bead foaming of poly(butylene terephthalate) by underwater pelletizing. J. Cell. Plast..

[28] Raps D., Köppl T., de Anda A.R., Altstädt V. (2014). Rheological and crystallisation behaviour of high melt strength polypropylene under gas-loading. Polymers (UK).

[29] Raps D., Köppl T., Heymann L., Altstädt V. (2011). Rheological behaviour of a high melt strength polypropylene at elevated pressure and gas-loading for foaming purposes. Rheol. Acta.

[30] Standau T., Zhao C., Murillo Castellón S., Bonten C., Altstädt V. (2019). Chemical modification and foam processing of polylactide (PLA). Polymers (Basel).

[31] Dorgan J.R., Lehermeier H., Mang M. (2000). Thermal and Rheological Properties of Commercial-Grade Poly(Lactic Acid)s. J. Polym. Environ..

[32] Yu L., Toikka G., Dean K., Bateman S., Yuan Q., Filippou C., Nguyen T. (2013). Foaming behaviour and cell structure of poly(lactic acid) after various modifications. Polym. Int..

[33] Takamura M., Sugimoto M., Kawaguchi S., Takahashi T., Koyama K. (2012). Influence of extrusion temperature on molecular architecture and crystallization behavior of peroxide-induced slightly crosslinked poly(L-lactide) by reactive extrusion. J. Appl. Polym. Sci..

[34] Soedergaard A., Niemi M., Selin J.F., Naesman J.H. (1995). Changes in Peroxide Melt-Modified Poly(L-lactide). Ind. Eng. Chem. Res..

[35] Engelberg I., Kohn J. (1991). Physico-mechanical properties of degradable polymers used in medical applications: A comparative study. Biomaterials.

