# Correction: Weingart et al. Expanded Polycarbonate (EPC)—A New Generation of High-Temperature Engineering Bead Foams. *Polymers* 2020, *12*, 2314

**DOI:** 10.3390/polym14101991

**Published:** 2022-05-13

**Authors:** Nick Weingart, Daniel Raps, Justus Kuhnigk, Andreas Klein, Volker Altstädt

**Affiliations:** 1 Department of Polymer Engineering, University of Bayreuth, 95444 Bayreuth, Germany; nick.weingart@uni-bayreuth.de (N.W.); justus.kuhnigk@uni-bayreuth.de (J.K.); 2 Covestro Deutschland AG, 51365 Leverkusen, Germany; daniel.raps@covestro.com (D.R.); andreas.klein@covestro.com (A.K.)

**Error in Table**

In the original publication [1], there was a mistake in the legend for Table 4: Summary of bending characteristic-values of EPC, EPP and EPET at 25, 80 and 110 °C. There is a copy and paste error. In the EPC 110 °C column, the values need to be (top to bottom) 61.0 ± 2.2; 1.4 ± 0.10; 9.5 ± 0.5; 7.7 ± 0.6. The correct legend appears below. The authors apologize for any inconvenience caused and state that the scientific conclusions are unaffected. The original publication has also been updated.

## Figures and Tables

**Table 4:** Summary of bending characteristic-values of EPC, EPP and EPET at 25, 80 and 110 °C.

		EPC	EPP	EPET
**25 °C**	**Bending-Modulus/MPa**	70.5 ± 4.5	45.3 ± 1.6	69.2 ± 6.6
	**Bending-Strength/MPa**	2.5 ± 0.20	1.60 ± 0.06	1.32 ± 0.05
	**Bending Strain at max. F/%**	7.7 ± 1.3	9.6 ± 0.7	2.2 ± 0.2
	**Elongation at max. F (Tensile)/%**	8.4 ± 1.2	20.2 ± 1.1	2.7 ± 0.4
		**EPC**	**EPP**	**EPET**
**80 °C**	**Bending-Modulus/MPa**	65.3 ± 3.0	8.3 ± 1.5	23.4 ± 1.5
	**Bending-Strength/MPa**	1.8 ± 0.12	0.54 ± 0.8	0.90 ± 0.03
	**Bending Strain at max. F/%**	8.0 ± 1.0	11.0 ± 0.5	5.5 ± 0.8
	**Elongation at max. F (Tensile)/%**	6.6 ± 0.5	51.0 ± 4.5	3.6 ± 1.1
		**EPC**	**EPP**	**EPET**
**110 °C**	**Bending-Modulus/MPa**	61.0 ± 2.2	3.0 ± 0.4	6.4 ± 0.8
	**Bending-Strength/MPa**	1.4 ± 0.10	0.25 ± 0.02	0.38 ± 0.04
	**Bending Strain at max. F/%**	9.5 ± 0.5	13.0 ± 0.8	7.6 ± 0.8
	**Elongation at max. F (Tensile)/%**	7.7 ± 0.6	97.3 ± 18.6	8.4 ± 0.3
